# Supplementary figures and images for: Additional predictors of stroke and transient ischaemic attack in BEFAST positive patients in out-of-hours emergency primary care
Source: PLoS One. 2024 Sep 20;19(9):e0310769. doi: 10.1371/journal.pone.0310769 (PMC11414940; doi:10.1371/journal.pone.0310769)

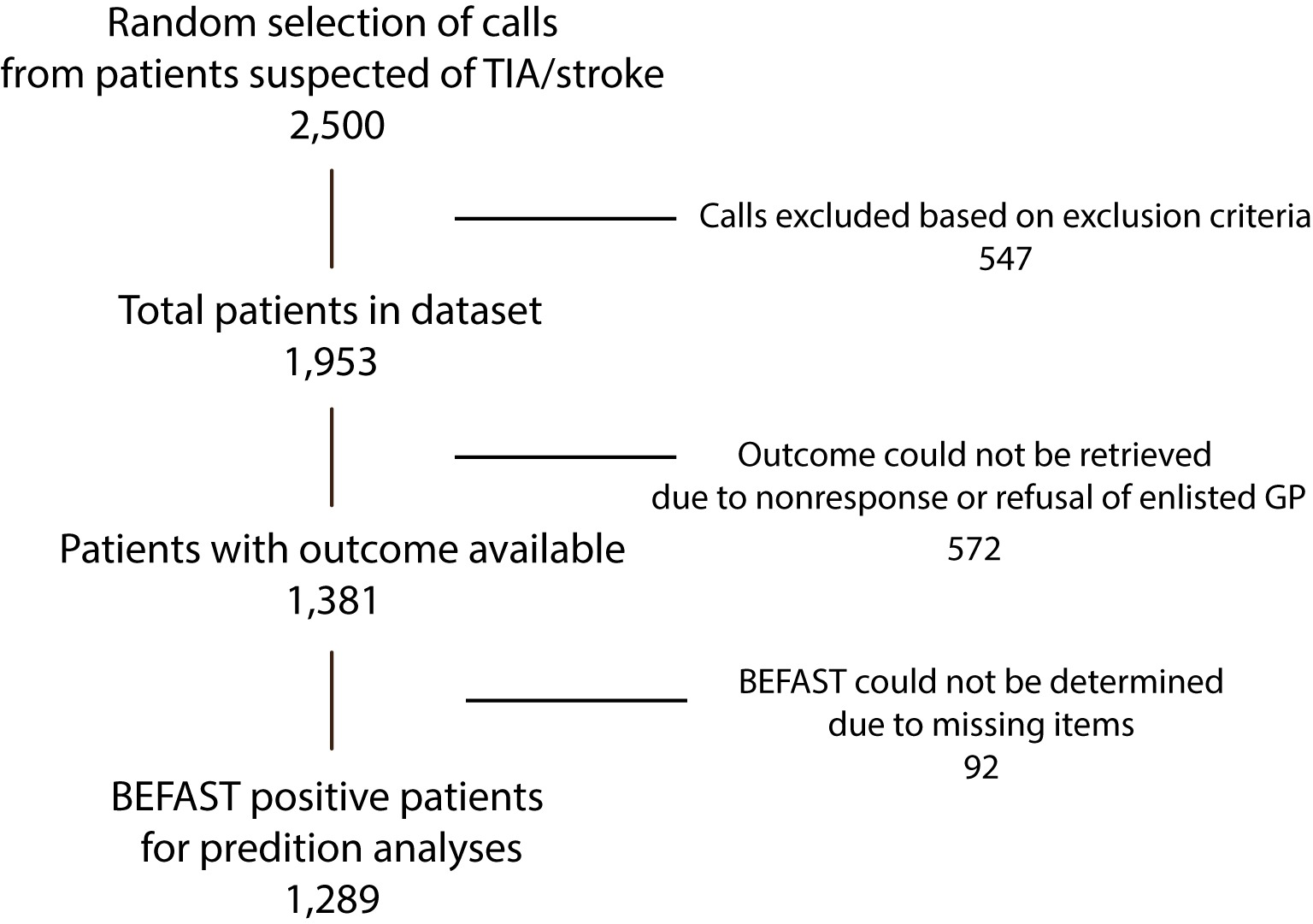

Supplement: S1 Fig — (TIF) [file pone.0310769.s001.tif]
