# Supplementary material for: Additional predictors of stroke and transient ischaemic attack in BEFAST positive patients in out-of-hours emergency primary care
Source: PLoS One. 2024 Sep 20;19(9):e0310769. doi: 10.1371/journal.pone.0310769 (PMC11414940; doi:10.1371/journal.pone.0310769)
Supplement: S1 Table — ICPC = International Classification of Primary Care. (DOCX) [file pone.0310769.s002.docx]

**S1 Table. ICPC codes, keywords and definition of BEFAST items used in this study.**

| **ICPC codes** | K89 (TIA), K90 (stroke), N17 (vertigo), N18 (neurological deficit), N19 (speech impairment), N29 (other neurological symptoms), N89 (migraine), N91 (facial paresis)) |
| --- | --- |
| **Keywords** | Neurological deficit, arm/leg weakness, face drooping, speech impairment, visual symptoms, sensory disturbances and common synonyms |
| **B** | Reporting of any gait or balance disturbances including leg weakness, vertigo and ataxia |
| **E** | Reporting of any visual symptoms such as vision loss and diplopia |
| **F** | Reporting of face drooping |
| **A** | Reporting of arm weakness in one or two arms |
| **S** | Reporting of any speech problem such as dysarthria or aphasia |
| **T** | Time from symptom onset (as an indication of treatment options if stroke or TIA is diagnosed, T was not included in the analyses) |

ICPC = International Classification of Primary Care
