# Supplementary material for: Additional predictors of stroke and transient ischaemic attack in BEFAST positive patients in out-of-hours emergency primary care
Source: PLoS One. 2024 Sep 20;19(9):e0310769. doi: 10.1371/journal.pone.0310769 (PMC11414940; doi:10.1371/journal.pone.0310769)
Supplement: S2 Table — (DOCX) [file pone.0310769.s003.docx]

**S2 Table. Percentage of missing data per predictor in BEFAST positive patients.**

| **Predictor** | **Percentage of missing data** |
| --- | --- |
| Age | 0% |
| Sex | 0% |
| History of CVD | 31% |
| History of TIA | 49% |
| History of stroke | 49% |
| Hypertension | 67% |
| Hypercholesterolaemia | 69% |
| Diabetes | 67% |
