# Supplementary material for: Additional predictors of stroke and transient ischaemic attack in BEFAST positive patients in out-of-hours emergency primary care
Source: PLoS One. 2024 Sep 20;19(9):e0310769. doi: 10.1371/journal.pone.0310769 (PMC11414940; doi:10.1371/journal.pone.0310769)
Supplement: S3 Table — Regression coefficients with 95% confidence intervals, c-statistic, and apparent performance measures of model 2, model 3 and model 4. Age was divided into three subgroups (shown as age, age’ and age”) using restricted cubic spline function to account for non-linearity. *The predictor history of cardio- and cerebrovascular disease is a combination of history of stroke, history of TIA and history of cardiovascular disease. **The predictor cardiovascular risk factors is a combination of diabetes, hypercholesterolaemia and hypertension. CI = confidence interval. (DOCX) [file pone.0310769.s004.docx]

**S3 Table. Model development and apparent performance of model 2, model 3 and model 4 using multivariable logistic regression.**

|  | **Model 2** |  | **Model 3** |  | **Model 4** |  |
| --- | --- | --- | --- | --- | --- | --- |
| **Predictor** | *Regression coefficient* | *95% CI* | *Regression coefficient* | *95% CI* | *Regression coefficient* | *95% CI* |
| **Intercept** | -5.946 | -8.481; -3.411 | -5.973 | -8.510; -3.436 | -5.946 | -8.481; -3.410 |
| **Age** | 0.112 | 0.060; 0.165 | 0.114 | 0.061; 0.166 | 0.113 | 0.060; 0.165 |
| **Age’** | -0.110 | -0.184; -0.035 | -0.110 | -0.185; -0.036 | -0.110 | -0.184; -0.035 |
| **Age’’** | 0.690 | 0.128; 1.252 | 0.694 | 0.131; 1.256 | 0.689 | 0.126; 1.251 |
| **Female sex** | 2.235 | -0.843; 5.313 | 2.269 | -0.810; 5.349 | 2.221 | -0.862; 5.304 |
| **Interaction sex and age** | -0.063 | -0.127; 0.001 | -0.064 | -0.128; 0.00 | -0.063 | -0.127; 0.002 |
| **Interaction sex and age’** | 0.113 | 0.019; 0.207 | 0.114 | 0.020; 0.208 | 0.113 | 0.019; 0.207 |
| **Interaction sex and age’’** | -0.636 | -1.353; 0.081 | -0.640 | -1.357; 0.077 | -0.635 | -1.353; 0.082 |
| **History of cardio- and cerebrovascular disease*** | 0.160 | -0.164; 0.484 |  |  | 0.203 | -0.439; 0.846 |
| **Cardiovascular risk factors**** |  |  | 0.113 | -0.218; 0.443 | -0.051 | -0.701; 0.599 |
|  | *Performance measures* | *95% CI* | *Performance measures* | *95% CI* | *Performance measures* | *95% CI* |
| **Apparent c-statistic** | 0.73 | 0.70; 0.75 | 0.73 | 0.70; 0.75 | 0.73 | 0.70; 0.75 |

Regression coefficients with 95% confidence intervals, c-statistic, and apparent performance measures of model 2, model 3 and model 4. Age was divided into three subgroups (shown as age, age’ and age”) using restricted cubic spline function to account for non-linearity. *The predictor history of cardio- and cerebrovascular disease is a combination of history of stroke, history of TIA and history of cardiovascular disease. **The predictor cardiovascular risk factors is a combination of diabetes, hypercholesterolaemia and hypertension. CI = confidence interval.
